# Pattern and prevalence of rifampicin and isoniazid-resistant tuberculosis using genotype MTBDR*plus* assay in Ethiopia

**DOI:** 10.1128/spectrum.01454-25

**Published:** 2026-02-20

**Authors:** Muluwork Getahun, Gobena Ameni, Hilina Mollalign, Getu Diriba, Dereje Beyene

**Affiliations:** 1 Ethiopian Public Health Institute 128164, Addis Ababa, Ethiopia; 2 Aklilu Lemma Institute of Pathobiology, Addis Ababa University 37602, Addis Ababa, Ethiopia; 3 Department of Veterinary Medicine, College of Agriculture and Veterinary Medicine, United Arab Emirates University 11239, Al Ain, UAE; 4 Department of Microbial, Cellular and Molecular Biology, Addis Ababa University 37602, Addis Ababa, Ethiopia; End TB Dx Consulting LLC, San Diego, California, USA

**Keywords:** drug-resistant survey, inferred mutation, treatment history, line probe assay, *Mycobacterium tuberculosis*, drug resistance mutations, molecular diagnostics

## Abstract

Resistance-conferring mutations in *Mycobacterium tuberculosis* (*Mtb*) are primary drivers of therapeutic challenges. However, comprehensive resistance mutation profiles are lacking in low- and middle-income countries. This study assesses the patterns and prevalence of isoniazid (INH) or multidrug/rifampicin-resistant (MDR/RR) tuberculosis (TB) mutations. We used stored *Mtb* isolates obtained from smear-positive TB patients recruited from 32 health facilities as part of the drug resistance survey (DRS). Line probe assay (LPA) testing was conducted on phenotypically confirmed INH-resistant and MDR/RR *Mtb* isolates. A total of 65 MDR/RR and 62 mono-INH-resistant *Mtb* isolates were analyzed. LPA detects 93.8% of rifampicin-conferred mutations in phenotypically confirmed MDR/RR *Mtb* isolates. The most frequent mutations were found at *rpoB* codons 530–533 (63.9%), and the S531L mutation comprised 59% of the MDR/RR TB isolates. The *katG*315 mutations were observed in the majority of MDR/RR (98%) and mono-INH-resistant (91.6%) *Mtb* isolates. The proportion of *katG*315 mutations was higher in newly diagnosed INH-resistant TB patients (72.7%) than those who had had prior treatment (27.3%). In 29.5% of MDR/RR and 2.5% of mono-INH resistant *Mtb* isolates, resistance was inferred. This study reports the proportion of mutations conferring resistance to rifampicin and INH using isolates collected from the national DRS.

**IMPORTANCE:**

Drug resistance mutations vary by location, effectiveness of the national control programs, and the diagnostic methods employed. Rapid molecular diagnostic tests are the primary methods used to detect drug-resistant tuberculosis. Comprehensive resistance mutation profiles are often lacking in low- and middle-income countries. The goal of this study was to assess the patterns and frequencies of mutations conferring first-line drug resistance in Ethiopia using isolates collected from the drug resistance survey. The isolates were obtained before the implementation of rapid molecular tests. The findings will enhance our understanding of the patterns and frequencies of mutations that confer resistance, which is crucial for developing a comprehensive catalog of mutations.

## INTRODUCTION

Tuberculosis (TB) and drug-resistant TB continue to pose a public threat (1). The World Health Organization estimated that nearly half a million people developed resistance to rifampicin TB. The estimated proportion of multidrug/rifampicin-resistant (MDR/RR)-TB among newly diagnosed TB cases is about 3–4%, while it is around 18–21% in previously treated TB patients (2). In Ethiopia, TB remains a public health issue, creating a significant burden on the health system (3). Ethiopia is 1 of the 30 high-burden countries for TB and human immunodeficiency virus co-infected TB patients, and it has been listed as 1 of the 30 high-burden MDR/RR TB countries for several decades (2, 3).

Resistance-conferring mutations vary by location, drug type, bacterial genetic background, anti-TB treatment history, and fitness cost (4–9). Additionally, the prevalence of resistance-conferring mutations could be influenced by the test coverage and the methods used (2, 10–12). Rifampicin and isoniazid (INH) are the key first-line drugs that guide the treatment decision (13). Rapid molecular diagnostic tests are the primary methods used to detect drug-resistant TB in low- and middle-income countries; however, the mutation catalogs in these regions are not comprehensive (11, 12).

The distribution of rifampicin and INH resistance-conferring mutations varies by region (4, 9, 14). Rifampicin resistance is mainly due to mutations in the hot-spot region of the *rpoB* gene (15). The three most common codons associated with *rpoB* mutations—531, 526, and 516—exhibit varying frequencies (16). Mutations at codon 531 are the most prevalent in various countries: India (55.6%), South Africa (91.8%), and Zambia (55.6%) (17–19). On the contrary, in Burkina Faso, the most common mutation occurred at codon 516 (51.5%) (14), while in Malawi, mutations at codon 526 (38%) were more frequently observed (20). Mutations in the *katG* gene are identified as the primary mechanism conferring resistance to INH (16). The proportion of *katG* mutations among MDR TB patients is higher than that observed in INH-resistant TB alone (21–23). Therefore, mutation catalogs are essential for informing the TB program on the optimal utilization of molecular diagnostic tools.

In Ethiopia, the Xpert MTB/RIF assay is the primary molecular diagnostic method employed for the diagnosis of drug-resistant TB. The line probe assay (LPA) has been used to detect drug-resistant TB at regional laboratories. Here, we use LPA to identify drug resistance-conferring mutations. The present study utilized strains collected throughout the country for the national drug resistance survey (DRS). The goal of our study is to assess the patterns and frequency of rifampicin and INH mutations.

## MATERIALS AND METHODS

### Study populations and settings

**Fig 1:**
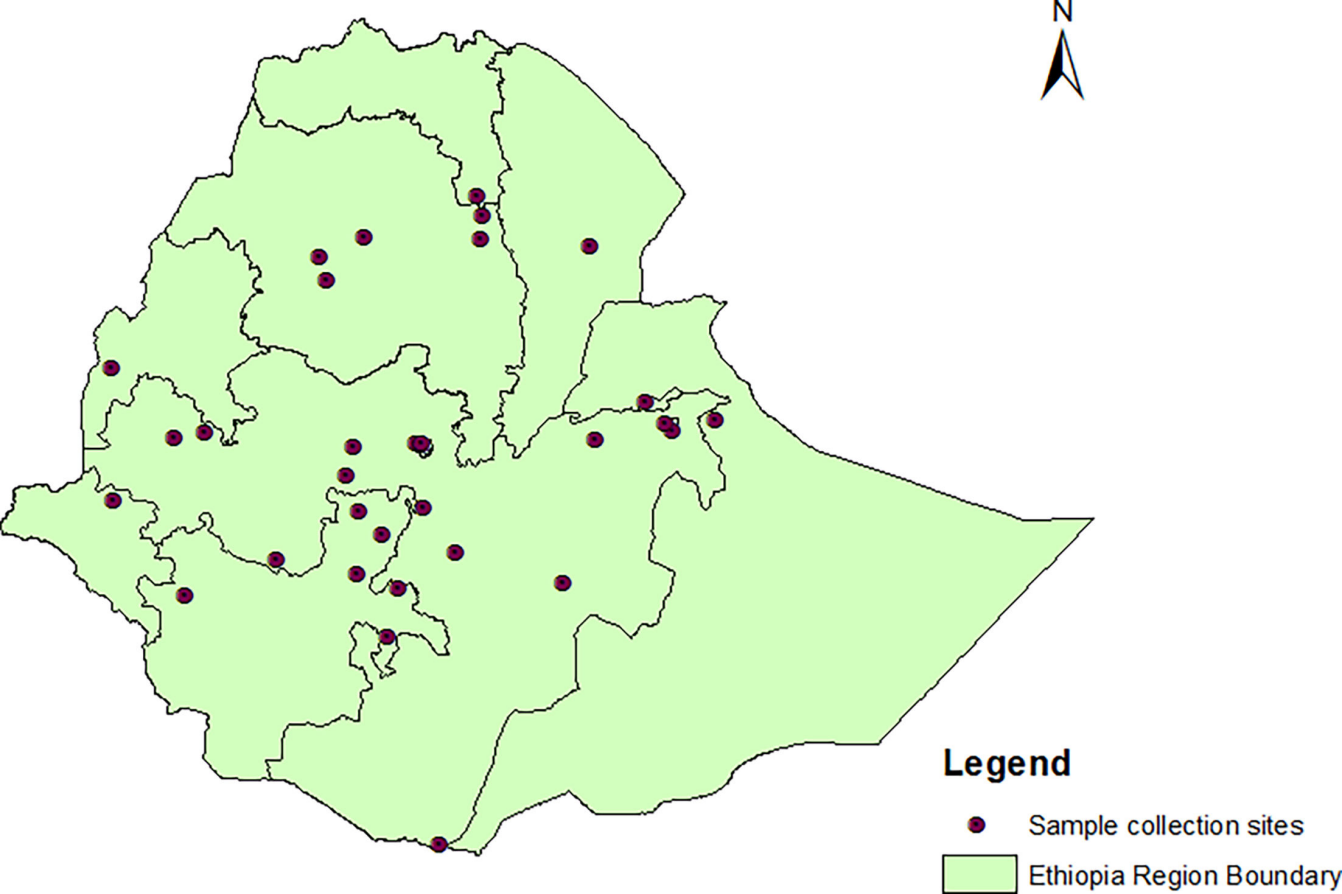
Distribution of sample collection sites.

**Fig 2:**
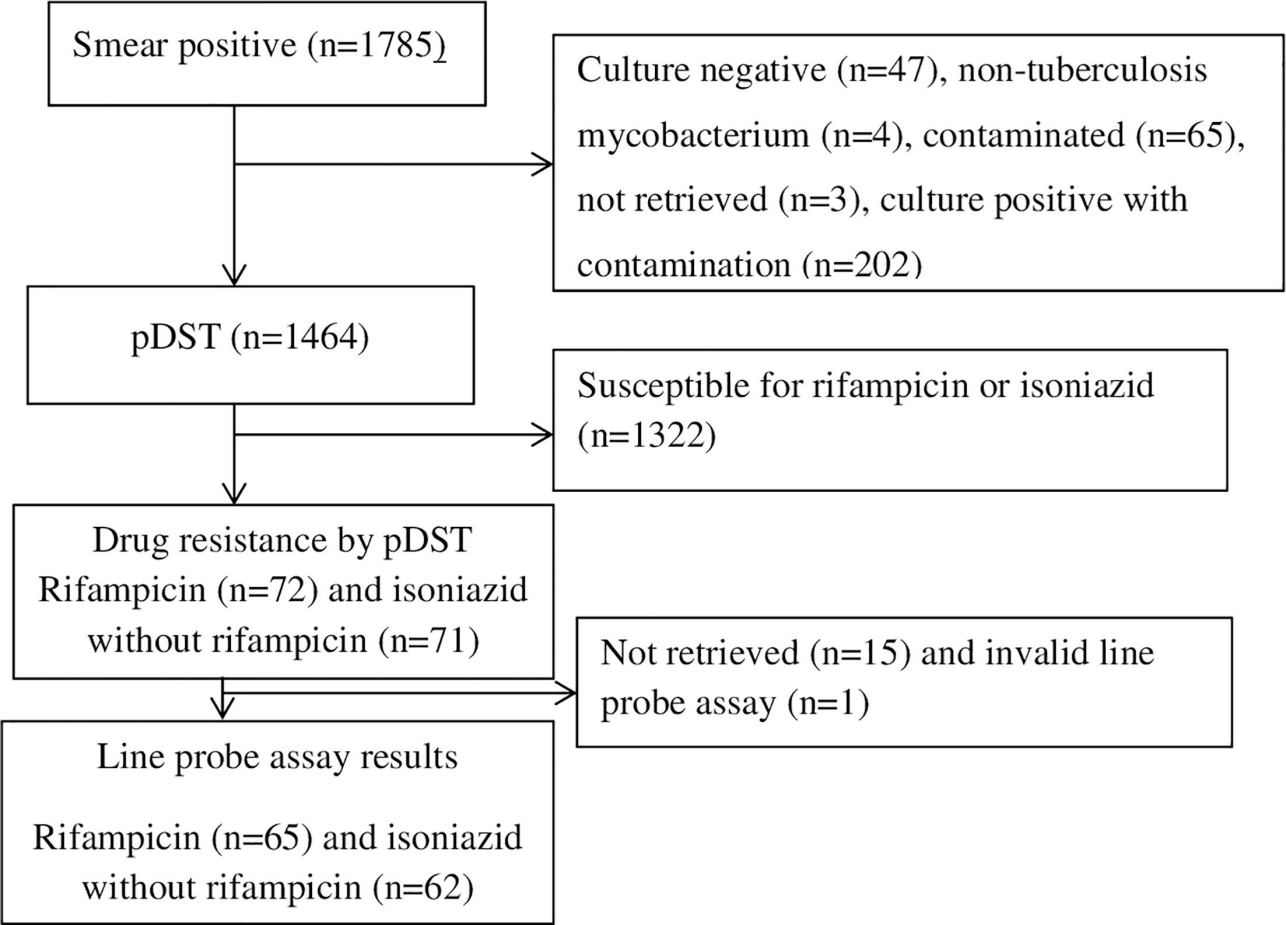
The number of isolates with phenotypic drug susceptibility test results for drug resistance survey and number of rifampicin and isoniazid resistance isolates characterized using line probe assay.

The study utilized *Mycobacterium tuberculosis complex* (*MTBC*) isolates collected for Ethiopia’s DRS. The DRS was conducted between November 2011 and June 2013 at 32 health facilities. Thirty of the 32 health facilities had been part of a previous DRS (2003–2005), and the other two were selected from regions that had not been covered before. The sample collection sites are displayed in Fig. 1. The target population of the DRS was newly diagnosed smear-positive TB patients. Previously treated smear-positive TB cases were also enrolled; however, they were not taken into account when determining the sample size. All consecutive sputum smear-positive TB cases were included in the DRS. A total of 1,785 smear-positive TB cases were enrolled for the survey, and pDST (phenotypic drug susceptibility testing) using Löwenstein-Jensen medium was available for 1,464 isolates. This study characterized phenotypically confirmed MDR/RR and INH resistance using LPA. The flowchart (Fig. 2) shows the number of MDR/RR and INH-resistant isolates characterized by LPA.

### Inclusion and exclusion criteria for DRS

- Inclusion criteria: The survey included all smear-positive TB cases irrespective of their age based on their consent.
- Exclusion criteria: Smear negative, extrapulmonary TB, and patients on anti-TB treatment during the intake period.

### LPA

After thawing frozen *Mtb* isolates, the bacterial suspensions were vortexed. We took 500 µL of the bacterial suspension and performed deoxyribonucleic acid (DNA) extraction using the GenoType MTBDR*plus* (Hain Lifescience, Germany, version 2) assay. Amplification, detection, and result interpretation were performed according to the manufacturer’s instructions. Briefly, the PCR was done in a final volume of 50 µL containing 10 µL of AM-A, 35 µL of AM-B, and 5 µL of the DNA supernatant. The amplification was done at 95°C for 15 min, followed by 10 cycles at 95°C for 30 s and at 65°C for 2 min. Next, it was subjected to 20 cycles comprised of 95°C for 25 s, 50°C for 40 s, and 70°C for 40 s, and then a final extension at 70°C for 8 min. Hybridization was performed with TwinCubator. After hybridization, the strips were fixed on the result interpretation paper. The results were interpreted if the three control bands (conjugate, amplification, and *MTBC*) and the three specific drug locus controls (*rpoB, katG*, and *inhA*) were present. The result interpretations were classified as follows: resistance not detected if all wild-type bands were present and no mutation band detected; resistance detected if any MUT band was detected; resistance inferred if one or more wild-type bands were missing without the corresponding MUT bands; and heteroresistance if both wild-type and resistance-conferring mutations were detected in a single isolate.

### Data analysis

Data were analyzed using SPSS and Stata. The frequency and percentage, along with a 95% confidence interval, were employed to describe the mutation profiles of rifampicin- and INH-resistant TB. *P* value and Cohen’s *h* were applied to assess the proportion of the *katG* mutations based on anti-TB treatment history. *P* values of less than 0.05 were considered statistically significant. Cohen’s *h* value of 0.8 and above was taken as a large effect.

## RESULTS

A total of 127 isolates from unique patients including 61 MDR, 4 RR, and 62 INH-resistant TB were analyzed. Rifampicin-conferring mutations were found or inferred by LPA in 61 (93.8%) of MDR/RR TB isolates. In 58 (95.1%) of MDR and 60 (96.7%) of mono-INH resistant *Mtb* isolates, LPAs were identified or inferred INH-conferring mutations.

### Mutation profiles of rifampicin-resistant TB

Ninety percent of the mutations were observed at codons 530–533 (63.9%) and 525–529 (26.2%). Resistance was confirmed in the majority (70.5%) of the isolates, and the three most frequent confirmed mutations were S531L (59%), H526Y (6.5%), and H526D (3.3%). Resistance was inferred in 29.5% of MDR/RR TB (Table 1).

**TABLE 1:** Rifampicin mutation profiles in multi-drug and isoniazid-resistant TB^*a*^

Resistant	Codon	Mutation band present (amino acid change)	Frequency	Percent	95% CI
Any RIF (*n* = 61)	505–509	Unknown	1	1.64	0.04–8.80
	513–519	Unknown	3	4.92	1.03–13.71
	513–519	MUT1 (D516V)	1	1.64	0.04–8.80
	516–519 and 525–529	Unknown	1	1.64	0.04–8.80
	525–529	Unknown	10	16.39	8.15–28.09
	525–529	MUT2A (H526Y)	4	6.56	1.82–15.95
	525–529	MUT2B (H526D)	2	3.28	0.40–11.35
	530–533	Unknown	3	4.92	1.03–13.71
	530–533	MUT3 (S531L)	35	57.38	44.06–69.96
	530–533	MUT3 (S531L)_het	1	1.64	0.04–8.80
MDR (*n* = 58)	505–509	Unknown	1	1.72	0.04–9.24
	513–519	Unknown	3	5.17	1.08–14.38
	513–519	MUT1 (D516V)	1	1.72	0.04–9.24
	516–519 and 525–529	Unknown	1	1.72	0.04–9.24
	525–529	Unknown	8	13.79	6.15–25.38
	525–529	MUT2A (H526Y)	4	6.90	1.91–16.73
	525–529	MUT2B (H526D)	2	3.45	0.42–11.91
	530–533	Unknown	3	5.17	1.08–14.38
	530–533	MUT3 (S531L)	34	58.62	44.93–71.40
	530–533	MUT3 (S531L)_het	1	1.72	0.04–9.24
RR (*n* = 3)	525–529	Unknown	2	66.67	0.84–90.57
	530–533	MUT3 (S531L)	1	33.33	9.43–99.16

^*a*^ het, heteroresistance; MDR, multidrug resistance; RIF, rifampicin; RR, rifampicin resistance; Unknown-mutation, not detected but inferred.

### Mutation profiles of INH-resistant TB

The *katG*315 mutations were observed in the majority of MDR/RR (98%) and mono-INH-resistant (91.6%) *Mtb* isolates. Resistance was confirmed in most (97.5%) of the isolates, and *katG* S315T1 was the highest (90.7%) of all. Resistance was inferred in a small fraction (2.5%) of any INH-resistant TB (Table 2). There was no significant difference in the proportion of *katG315* between MDR patients who had previously received treatment (57.9%) and those who were newly diagnosed (42.1%) (*P* = 0.092, Cohen’s *h* value = 0.3169). However, newly diagnosed (72.7%) INH-resistance and rifampicin-susceptible (INH_R_RIF_S_) TB patients had a considerably greater proportion of *katG315* mutations than previously treated (27.3%) individuals, with a large effect size (*P* < 0.001, Cohen’s *h* value = 0.9437). Most of the newly diagnosed INH_R_RIF_S_ TB patients (72.3%) had additional drug resistance to ethambutol or streptomycin (Table 3).

**TABLE 2:** Isoniazid mutation profiles in multi-drug and isoniazid-resistant TB^*a*^

Resistant profile	Codon	Mutation band present (amino acid change)	Frequency	Percent	95% CI
Any INH (*n* = 118)	315	MUT1 (S315T1)	97	82.20	74.09–88.63
	315	MUT1 (S315T1)_het	10	8.47	4.14–15.03
	315	Unknown	3	2.54	0.53–7.25
	315	MUT2 (S315T2)	1	0.85	0.02–4.63
	315 and −15	MUT1 (S315T1) and MUT1 (c-15t)	1	0.85	0.02–4.63
	−15	MUT1 (c-15t)	6	5.08	1.89–10.74
INH (*n* = 60)	315	MUT1 (S315T1)	43	71.67	58.56–82.55
	315	MUT1 (S315T1)_het	9	15.00	7.10–26.57
	315	Unknown	1	1.67	0.04–8.94
	315	MUT2 (S315T2)	1	1.67	0.04–8.94
	315&−15	MUT1 (S315T1) and MUT1 (c-15t)	1	1.67	0.04–8.94
	−15	MUT1 (c-15t)	5	8.33	2.76–18.39
MDR (*n*=58)	315	MUT1 (S315T1)	54	93.10	83.27–98.09
	315	MUT1 (S315T1)_het	1	1.72	0.04–9.24
	315	Unknown	2	3.45	0.42–11.91
	−15	MUT1 (c-15t)	1	1.72	0.04–9.24

^*a*^ het, heteroresistance; INH, isoniazid; MDR, multidrug resistance; Unknown-mutation, not detected but inferred.

**TABLE 3:** Proportion of *katG* mutation at codon 315 by drug profiles and history of anti-tuberculosis treatment^*a*^

DST results	Mutation at *katG*	Newly diagnosed TB patient			Previously treated TB patient			*P* value	Cohen’s *h*
		Frequency	Percent	95% CI	Frequency	Percent	95% CI		
MDR	57	24	42.11	29.14–55.92	33	57.89	44.08–70.85	0.092	0.3169
INH without RIF	55	40	72.72	59.04–83.86	15	27.27	16.14–40.96	<0.001	0.9437
INH only	8	6	75.00	34.91–96.81	2	25.00	3.18–65.08	0.046	1.0472
INH+SM/EMB	47	34	72.34	57.36–84.37	13	27.66	15.62–42.63	<0.001	0.9264

^*a*^ EMB, ethambutol; INH, isoniazid; MDR, multidrug resistance; RIF, rifampicin; SM, streptomycin.

## DISCUSSION

This work provides the pattern and prevalence of mutations associated with rifampicin and INH resistance using *Mtb* isolates collected from DRS. We report the proportion of prevalent and uncommon mutations associated with rifampicin and INH resistance.

Mutations primarily causing rifampicin resistance occurred in the hot-spot region of the *rpoB* gene (24). S531L was the most frequent mutation, and it varies by country. The prevalence of S531L in Uganda is 40%, while in Kazakhstan, it is 87% (4, 5). In our study, mutation at S531L accounted for 59% of MDR/RR-TB. A similar high proportion of S531L mutation has been reported in Oman (53%), Europe (50.9%), and China (56.7%) (21, 25, 26). In contrast, the prevalence in Kazakhstan (87.4%), South Africa (62–91.8%), and Rwanda (86%) is substantially higher (4, 11, 18, 27). Epistasis interactions between the mutations and strain genetic backgrounds partly influence the proportion of S531L (7, 28). Lineage 4 is prevalent in Ethiopia, while lineage 2 was more prevalent in South Africa and Kazakhstan (4, 29, 30). Low-cost mutations like S531L are more likely to be found in lineage 2 than lineage 4 (7). On the contrary, a higher prevalence of S531L has been detected in Rwanda than in Ethiopia, even though lineage 4 is the predominant lineage in both countries (11). This might be the result of strain clustering rate and the diagnostic technique employed. In Rwanda, a single dominant clone was found in 69.1% of all MDR/RR-TB cases (82.2% of clustered), and 93.8% of MDR/RR-TB cases had the S531L mutation when pDST was the only detection method (11, 31).

Most mutations that cause INH resistance are associated with the *katG* gene (32, 33). The *katG*315 mutation was the most prevalent mutation (64%) among phenotypic INH resistance. *katG* S315T was the most prevalent nucleotide change of *katG*315 substitution (23, 34). We found a higher proportion of *katG*315 in INH_R_RIF_S_ TB patients who had been newly diagnosed, and most (72%) of them had additional drug resistance, which indicates that a higher proportion of *katG*315 was likely due to transmission (35, 36). Additionally, our results showed that there is no difference in the percentage of *katG315* mutations between patients with MDRTB who have had prior treatment and those who have not. *katG* mutation has been shown to be highly prevalent among MDRTB in both high- and low-MDR-burden countries (21, 22). Furthermore, the association of *katG* mutation with previously treated TB patients has been reported in a couple of studies (9, 37, 38). The disparity with our findings may be due to the high occurrence of the *katG315* mutation in newly diagnosed TB patients, which is expected when there is limited access to diagnostic methods for drug resistance. In Ethiopia, at the time of the survey, the drug susceptibility testing was only available at the National Reference Laboratory.

MTBDR*plus* identifies rifampicin-associated mutations using four *rpoB* probes (Asp516Val, His526Asp, His526Tyr, and Ser531Leu). Additionally, INH-associated mutations can be detected through two *katG* (Ser315Thr) and two *inhA* probes (*inhA* promoter mutations). The remaining mutations are inferred from the absence of a wild-type probe (39, 40). In our findings, resistance was inferred in 29.5% of rifampicin-resistant and 2.5% of INH-resistant TB, which is in line with previous studies in Ethiopia (41, 42). Similar observations have been reported from other countries (5, 18). The prevalence of inferred mutations is common in certain countries, and those mutations were classified as borderline resistance in most instances (5, 43, 44). Borderline resistances are often misdiagnosed when using mycobacterium growth indicator testing; however, they have been identified through molecular methods (11, 44). The findings from Rwanda and China indicate an increase in the detection of borderline RR-associated mutations through the use of whole genome sequencing or Xpert MTB/RIF assays, which have been misdiagnosed by pDST (11, 45). Overall, understanding the patterns and frequencies of resistance-conferring mutations is crucial to developing a comprehensive mutation catalog particularly for key anti-TB drugs, which play a significant role in assay design (46). Therefore, an epidemiological study aimed at identifying mutations that confer resistance can enrich a regional mutations catalog which is essential to improving the accuracy of resistance predication.

### Conclusion

We report the patterns and frequencies of mutations associated with rifampicin and INH resistance using isolates for the DRS survey that were collected before the wide implementation of rapid molecular techniques. The proportion of resistant TB with mutations inferred suggests that the prevalence of those mutations was not rare.

## Data Availability

All information pertaining to this manuscript is included in this paper. The raw data has been archived at the Ethiopian Public Health Institute.
